# CRABR-Net: A Contextual Relational Attention-Based Recognition Network for Remote Sensing Scene Objective

**DOI:** 10.3390/s23177514

**Published:** 2023-08-29

**Authors:** Ningbo Guo, Mingyong Jiang, Lijing Gao, Yizhuo Tang, Jinwei Han, Xiangning Chen

**Affiliations:** 1 Space Information Academic, Space Engineering University, Beijing 101407, China; sxguonb@163.com (N.G.);; 2 State Key Laboratory of Remote Sensing Science, Aerospace Information Research Institute, Chinese Academy of Sciences, Beijing 100101, China

**Keywords:** attentional mechanisms, relationship feature, scene objective, feature integration

## Abstract

Remote sensing scene objective recognition (RSSOR) plays a serious application value in both military and civilian fields. Convolutional neural networks (CNNs) have greatly enhanced the improvement of intelligent objective recognition technology for remote sensing scenes, but most of the methods using CNN for high-resolution RSSOR either use only the feature map of the last layer or directly fuse the feature maps from various layers in the “summation” way, which not only ignores the favorable relationship information between adjacent layers but also leads to redundancy and loss of feature map, which hinders the improvement of recognition accuracy. In this study, a contextual, relational attention-based recognition network (CRABR-Net) was presented, which extracts different convolutional feature maps from CNN, focuses important feature content by using a simple, parameter-free attention module (SimAM), fuses the adjacent feature maps by using the complementary relationship feature map calculation, improves the feature learning ability by using the enhanced relationship feature map calculation, and finally uses the concatenated feature maps from different layers for RSSOR. Experimental results show that CRABR-Net exploits the relationship between the different CNN layers to improve recognition performance, achieves better results compared to several state-of-the-art algorithms, and the average accuracy on AID, UC-Merced, and RSSCN7 can be up to 96.46%, 99.20%, and 95.43% with generic training ratios.

## 1. Introduction

RSSOR is popularly adapted to specific tasks such as geological exploration, precision agriculture, and urban planning [1,2,3]. As the name implies, RSSOR infers the right category of scene objectives by evaluating the content features that are included in the remote sensing data. With the continuous advancement of urban construction and the rapid progress of high-resolution observation satellites, the characteristics of diverse feature objectives and the scale of data are increasing, and how to perform RSSOR more accurately is already a popular and difficult problem for ongoing research in the field of remote sensing technology development [4,5,6].

With the accumulation of data volume and the improvement of computer performance, artificial neural networks and deep learning networks are developing rapidly, and the use of CNN for RSSOR has come into being [7]. CNN, as one of the emerging artificial neural network technologies, merges intelligent deep learning techniques, and has the advantages of “sparse connection”, “parameter sharing”, and “equivariant representation” [8]. It can shorten the time required for model learning, lower the volumes of data requiring training parameters, and reduce the memory requirement for model operation. In addition, the feature maps obtained by using CNN generally have three layers: the bottom layer reflects the details of the color, texture, and shape of the objective; the middle layer reflects the state of an object in the image at a certain moment; and the top layer reflects the overall concept of the image with rich semantic information. In particular, it should be said that the top-layer feature maps are also the most applied in RSSORs. However, when CNN is employed for RSSOR, ignoring the other layers and just adopting the last layer not only fails to improve the recognition performance but also cannot fully exploit the advantages of CNN [9].

Another popular method based on CNN is to integrate the hessian eigenmaps learned from different CNN layers to generate new discriminative feature maps for RSSOR, which can achieve complementary feature advantages and even improve the recognition effect of the network. Two structures are common for multilayer feature fusion networks: the first is a parallel multi-branch network (PMBN), and the other is a serial hop-layer connection network (SHLCN). PMBNs are usually used to fuse features using different convolutional kernels, convolution with holes, and pooling operations of different sizes. In [10], the features are first extracted and then fused using four parallel structures, each containing convolutional kernels of different sizes. In [11], highly accurate features were obtained using convolutional networks with holes. In [12], the recognition accuracy of small samples is improved by assembling feature maps of different scales under different weights. The above methods achieve their purpose, but they ignore the relationship between adjacent layers. SHLCN is a combination of features implemented through hop-level connections. In [13], the fusion of features obtained by using layer-hopping connections for recognition is superior to traditional methods. In [14], the covariance matrix is obtained by superimposed multilayer features, and then the covariance matrix and support vector machine are used to further obtain better classification results. In [15], sparse representation is used to fuse the middle layer and top layer features, and then the fused features are used for scene classification, which is effective for classification in limited data. The above method utilizes multilayer feature fusion, but there are problems of feature redundancy and offset in the integration process, which also ignores the relationship between adjacent layers. In summary, it is easy to understand that the parallel structure is able to acquire different perceptual field features at the same level, while serial structures are able to integrate features from various levels. All these methods are able to enhance the features, but they also bring the problems of redundancy and mutual exclusion of feature maps.

In addition, because of the complex and diverse characteristics of the features themselves, the satellite will be affected by the background, lighting, scale, and other imaging conditions in the process of photography. Therefore, two types of feature confusion problems arose in RSSOR: scene objectives with similar semantic categories probably share different visual variability, and scene images of different semantic categories may also have certain similarities [16]. To reduce the impact of these two problems, many researchers have tried to use an attentional mechanism (AM) [17]. In [18], a dual-attention residual network is designed to extract features, embedding spatial attention into the bottom features and channeling attention into the top features. In [19], adding AM to top-level features, selectively focusing on key content, and discarding non-key information improves classification performance. The above methods only add attention features after convolutional processing, so that attention features can only be learned from the current feature layer, ignoring the attention relationship with other convolutional layers.

To fully exploit the powerful learning capability provided by CNNs while reducing the impact of feature confusion for remote sensing scene objective recognition, inspired by the literature [20] and AM, we plan to explore the complementary relationships and enhanced relationship messages existing between feature maps of adjacent convolutional layers, focusing on key messages and discarding non-key messages in the process of feature maps computation.

In general, this study has three main contributions.

(1) A complementary relational feature computation module is designed;
(2) An enhanced relational feature calculation module is designed;
(3) A contextual, relational attention-based recognition network is proposed to effectively enhance the performance of RSSOR using CNN.

Other important contents are organized as follows: Section 2 describes related work; Section 3 introduces CRABR-Net; Section 4 reports the experimental results; Section 5 carries out the discussion; Section 6, the paper is summarized.

## 2. Related Work

### 2.1. Methods Based on Intuitive Feature

This category is the earliest recognition method to identify the category of an image by the most intuitive underlying features of the scene objectives. The underlying features consist of local features and global features, such as color, spectrum, texture, structure, and so on [21]. Color features are typical local features, and they are also the most easily observed and calculated underlying features [22]. A common method of identifying color histograms is to interpret categories by comparing the proportions of different colors in the entire image [23]. This method cannot determine the spatial position of each color in the image, and is less effective in identifying images that are spectrally similar but have large differences in distribution. Texture features are a type of global feature [24]. Typical methods, such as de-identification using the grayscale covariance matrix, are used to calculate the gray-level covariance matrix of an objective, and then the categories are identified by analyzing the features of the image [25]. This method is more effective in recognizing images with large differences in texture features, but it is not easy to recognize scene images with insignificant texture features.

### 2.2. Methods Based on Statistical Features

This method is an agglutination or consolidation of intuitive features, and its essence is to analyze the statistical distribution of image intuitive features to establish the connection between them and semantic features, and the representative methods are bag of visual words (BoVW) and k-mean clustering methods [26]. The core idea of BoVW is to count the underlying features of an image, such as SIFTI [27], GIST [28], etc., and then analyze these underlying features by clustering methods such as K-mean to form a “visual dictionary”, and then encode the image according to the frequency of the intuitive features appearing in the “visual dictionary”, as a feature description of the image. The BoVW method recognizes better than the method based on intuitive features, but only utilizes the frequency information of the visual lexicon, ignores the spatial distribution relationship, and lacks the correlation between the features, which still has limitations. Later, there are some improved methods, such as spatial pyramid matching [29], to segment the image at multiple scales and enhance the spatial information. However, these methods still need to extract many intuitive features, which are not only cumbersome and inflexible, but also easy to ignore semantic information.

### 2.3. Methods Based on Depth Feature

These methods utilize deep learning models to adaptively learn objectives in an “end-to-end” manner, and achieve higher accuracy after obtaining deep semantic information. Commonly used models include Stacked Auto Encoder (SAE) [30], Visual Transformer (VIT) [31], CNN [32], etc. For example, Li et al. [33] will apply the SAE; the model is simpler, and the feature representation of the input data can be quickly established by a small number of features, but this type of method is unable to catch the spatial relationship among the local features. Bazi et al. [34] utilize VIT and achieve a high recognition accuracy, but these methods take a long time to train and need a large amount of Objective information to achieve a relatively good training result. Methods utilizing CNN are the most popular approaches for RSSOR [9]. Generally, according to the way of deep feature utilization, the method can be categorized into CNN without fusion method, CNN with fusion method, and CNN with AM method.

- *CNN without Fusion Method*. The method utilizes CNN to acquire local features of the training objectives and then transforms them directly into global features for recognition [35]. According to whether pretraining parameters are used or not, the present method can be categorized into two classes. One class does not use pretraining parameters. Nogueira et al. [36] apply popular CNNs, such as AlexNet, VGG, PatreoNet, etc., to RSSOR, respectively, and achieve good recognition results without pretraining parameters. Another category uses pretraining parameters. Castelluccio et al. [37] demonstrate the importance of adopting pretraining parameters for CNN by importing the pretraining parameters of CaffeNet and GoogLeNet and applying them to RSSOR, respectively;
- *CNN with Fusion Method.* The methods perform the fusion process on the features of CNN-extracted images. One class of methods utilizes a single CNN to extract features and then fuses them. Yuan et al. [38] directly stitche the last convolutional layer feature and the last fully connected layer feature of VGG-19 as the final representation of the image. Xu et al. [39] processed the convolutional features of layers 4, 7, 10, and 13 of VGG-16 and obtained converged features. The other is utilizing multiple CNNs to draw features, which are then fused. Zhang et al. [40] propose the use of multiple CNNs to extract local features of an image. Liu et al. [41] use CaffeNet and VGG-VD16 to extract deep features and then rearrange and combine them for recognition; Yu et al. [42] use three networks, CaffeNet and its improved network, and improved VGG network, to extract features and fuse them for recognition;
- *CNN with AM Method.* The methods usually add AM behind the convolutional layer to filter useless information and enhance useful features. For example, the literature [43] added a channel attention mechanism [44] to different stages of DenseNet-121, and Guo et al. [18] added a spatial attention mechanism [45] to the second convolutional module of ResNet-101, and channel attention to the third, fourth, and fifth convolutional modules. Wang et al. [19] propose a mask matrix as a convolutional feature for attention; Fan et al. [46] design an attention mechanism with trunk branches and mask branches for ResNet-50.

All of the above methods work well in RSSOR, but where these methods either utilize a certain layer of features or simply sum the features of several layers, ignoring the relational information between the features, our goal is to maximize the use of CNN extracted features of each layer, and to obtain a better recognition effect just by one CNN backbone network.

## 3. Methodology

**Figure 1:**
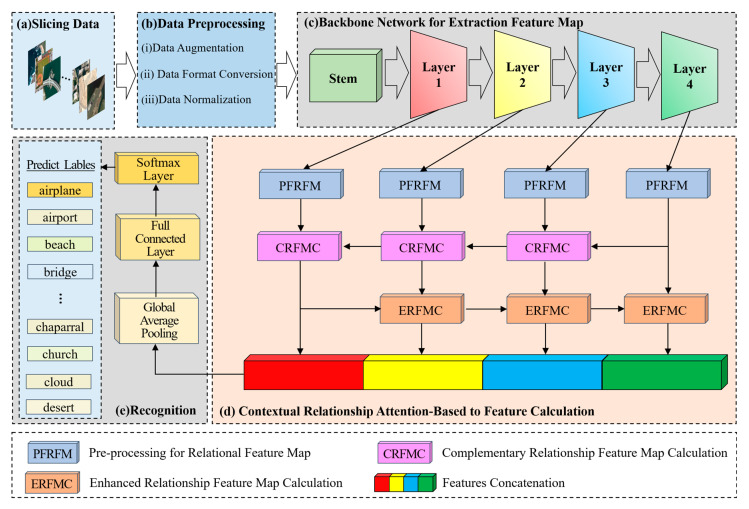
The general structure of CRABR-Net.

The architecture of the CRABR-Net proposed is shown in Figure 1. It contains 5 main steps.

**Figure 2:**
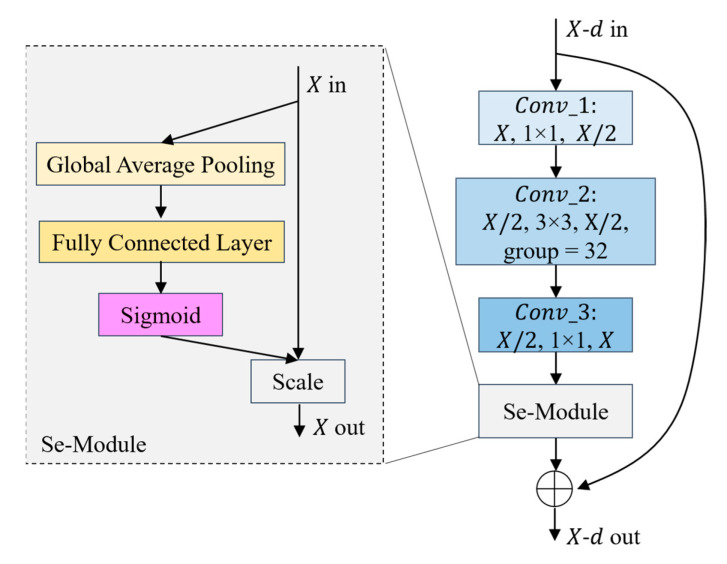
Example of the Bottleneck for Se-ResNeXt-50.

**Figure 3:**
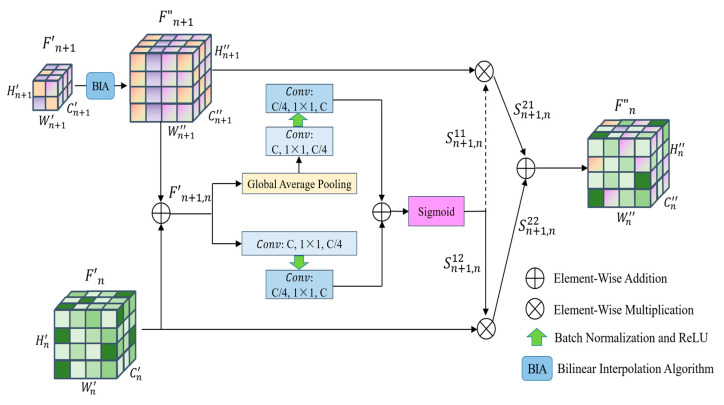
Complementary relationship feature map calculation.

**Figure 4:**
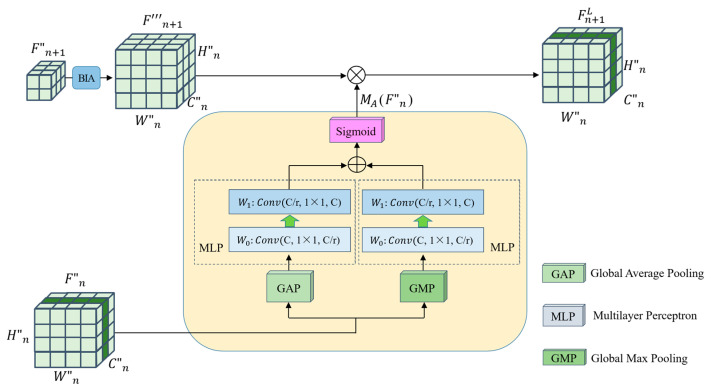
Enhanced relationship feature map calculation.

(a) The first step is to divide the data. Divide the remote sensing image dataset into the training dataset and verify the dataset according to a certain ratio (e.g., 4:1);
(b) The second step is data preprocessing. Firstly, augment the remote sensing image data to be input, including randomly cropping to 256 × 256, randomly rotating between −45 degrees and 45 degrees, flipping horizontally with 0.5 probability, and then cropping to 224 × 224; then converting the format, converting the data format to (Batch, Channel, Height, Width); and finally normalizing the data, setting the mean value of Height and Width of every Channel’s Height and Width mean value is set to 0 and standard deviation is set to 1, respectively;
(c) The third step is to extract features with the backbone network, a Bottleneck is shown in Figure 2. The parameters that have been trained on the Image-Net dataset [47] are imported into the Se-ResNext-50 network, the fully connected layers of the original network are replaced with the network structure designed in steps d and e, and then go on to extract $F_{1}$, $F_{2}$, $F_{3}$, $F_{4}$ of the four different convolutional layers;
(d) The fourth step is to compute the relationship enhancement features. ***(1) PFRFM***. Obtain the refined features ${F^{\prime }}_{1}$, ${F^{\prime }}_{2}$, ${F^{\prime }}_{3}$, ${F^{\prime }}_{4}$ by using SimAM. ***(2) CRFMC***. Sum the elements at the corresponding positions of ${F^{\prime }}_{4}$ and ${F^{\prime }}_{3}$ to obtain ${F^{\prime }}_{4,3}$. Before summing, up-sample ${F^{\prime }}_{4}$ by a factor of 2 to obtain ${F^{\prime \prime }}_{4}$. Similarly, we obtain ${F^{\prime }}_{3,2}$ and ${F^{\prime }}_{2,1}$. For ${F^{\prime \prime }}_{3}$, ${F^{\prime \prime }}_{2}$, ${F^{\prime \prime }}_{1}$, the processing flow shown in Figure 3 can be utilized by using ${F^{\prime }}_{4,3}$, ${F^{\prime }}_{3,2}$, ${F^{\prime }}_{2,1}$ respectively. ***(3) ERFMC***. For $F_{2}^{L}$, ${F^{\prime \prime }}_{1}$ is transformed into $Z_{GAP}({F^{\prime \prime }}_{1}\;)$ = [B, 256, 1, 1] and $Z_{GMP}({F^{\prime \prime }}_{1}\;)$ = [B, 256, 1, 1] using GAP and GMP, respectively, and then linearly transformed using MLP to obtain $M_{GAP}(Z_{GAP}({F^{\prime \prime }}_{1}\;))$ = [B, 256, 1, 1] and $M_{GMP}(Z_{GMP}({F^{\prime \prime }}_{1}\;))$ = [B, 256, 1, 1], respectively. $M_{A}({F^{\prime \prime }}_{1}\;)$ are obtained through Equation (9). Up-sampling ${F^{\prime \prime }}_{2}$ by a factor of 2 yields ${F^{\prime \prime \prime }}_{2}$, and multiplying ${F^{\prime \prime \prime }}_{2}$ by $M_{A}({F^{\prime \prime }}_{1}\;)$ yields $F_{2}^{L}$. Similarly, $F_{3}^{L}$ and $F_{4}^{L}$ can be obtained. Specifically, ${F^{\prime \prime }}_{1}$ equals $F_{1}^{L}$. The process is illustrated in Figure 4. ***(4) Feature Fusion**.* Using Equation (11), splice $F_{1}^{L}$, $F_{2}^{L}$, $F_{3}^{L}$, and $F_{4}^{L}$ to obtain F = [B, 1024, 56, 56];
(e) The fifth step is to recognize. F is fed into a recognizer consisting of GAP, Fully Connected Layer, and Softmax Layer for scene recognition.

### 3.1. Backbone Network for Extraction Feature Map

We use Se-ResNext-50 as the feature extraction backbone network for this remote sensing image recognition task. Se-ResNext-50 retains the advantages of the residual structure of ResNet, adopts ideas from the inception network model in widening network processing, and combines the advantages of the Se-Net network to exploit the relationship between channels between features, which performs better in feature learning compared to ResNet and other variants of the network [48].

As shown in © CNN Backbone Network in Figure 1, the Stem module, layer1 module, layer2 module, layer3 module, and layer4 module in the Se-ResNext-50 network are used to compute the preprocessed dataset in turn for obtaining the output feature maps from the four-level modules. Within the Stem module, 64 convolution kernels of size 7 × 7 are used for the convolution calculation at one step of 2. Then, the feature maps obtained in step 1 are pooled with a window of 3 × 3 and a maximum value of 2 for obtaining a feature mapping with a size variation of 56 × 56.

As shown in Figure 2, the Layer1 module contains three groups of Bottleneck. Each group of Bottleneck consists of Conv_1, Conv_2, Conv_3, and Se-Module, where the convolutional kernel sizes of the three convolutional modules are 1 × 1, 3 × 3, and 1 × 1, and the numbers of convolutional kernels are 128, 128 and 256, in that order. Specifically, in the second convolution stage, 32 identical structures are utilized to widen this network module. In this se-module, the compression is performed using global average pooling, followed by modeling associations between channels through a full connectivity layer, a sigmoid function to export weights with an equal amount of input features, and finally, the normalized weights are added onto the features per channel. Similar to the Layer1 module, the number of Bottleneck compositions of Layer2, Layer3, and Layer4 modules are 4, 6, and 3, respectively, and each Bottleneck consists of Conv_1, Conv_2, Conv_3, and Se-Module, and the number of convolutional cores are, respectively [256, 256, 512], [512, 512, 1024], [1024, 1024, 2048]. After the calculation of each module above, we obtained the feature maps of four different convolutional layers, which are $F_{1}$ = [B, 256, 56, 56], $F_{2}$ = [B, 512, 28, 28], $F_{3}$ = [B, 1024, 14, 14], and $F_{4}$ = [B, 2048, 7, 7].

### 3.2. Preprocessing for Relational Feature Map

To prevent the model from becoming more complex and to control the number of parameters as much as possible, we use SimAM [49] to focus the feature expressions of the four different layers deeper into the more important information without increasing the network parameters.

In order to facilitate the primary relational feature calculation and advanced relational feature calculation later, we use 1 × 1 convolution to perform channel reduction operation on the features maps. We design the convolutional dimensionality reduction module separately; the input size of the convolution kernel is set to the channel number scale of the input features, and the output number of the convolution kernel is kept the same as the channel number $F_{1}$.

In the above processing, to avoid the instability of the network learning process due to the oversized feature data after the convolutional dimensionality reduction calculation, we batch normalize the dimensionality reduction results so that the feature data satisfy the distribution law of mean 0 and variance 1. In addition, to avoid over-fitting, we add a modified linear function [50] to keep only the outputs larger than 0, and other inputs will be set to 0, so that the network can be better fitted.

So far, we obtained the results after relational feature maps preprocessing as ${F^{\prime }}_{1}$ = [B, 256, 56, 56], ${F^{\prime }}_{2}$ = [B, 256, 28, 28], ${F^{\prime }}_{3}$ = [ B, 256, 14, 14], ${F^{\prime }}_{4}$ = [B, 256, 7, 7].

### 3.3. Complementary Relationship Feature Map Calculation

Information about the relationship between ${F^{\prime }}_{1}$, ${F^{\prime }}_{2}$, ${F^{\prime }}_{3}$ and ${F^{\prime }}_{4}$ should be fully utilized. We design a primary relationship enhancement process from the high feature layer to the low feature layer to further extract the relationship between adjacent layer features and embed this relationship into the adjacent low layer features to complement the performance of low layer features, and the structure is described in Figure 3.

In aiming to utilize the adjacent high convolutional layers to complement the missing global message of low-level features, we enhance the size of high-level feature maps with a bilinear difference algorithm to match the size of the feature maps acquired from low-level convolutional layers. In particular, unlike the literature [20], considering various fusion methods of convolutional features from adjacent layers will have variable effects on integrated features; instead of simply using the direct summation of the corresponding elements, we obtain the primary relational features by assigning different weight parameters to the adjacent feature layers and then multiplying the corresponding elements with the weights before summation.

As seen in Figure 3, firstly achieve size augmentation of dimensions between relational features by a bilinear interpolation algorithm, and then the dimensionally augmented feature map and the underlying feature map in its adjacent layers are sequentially summed by the corresponding positions of the pixels to acquire the fused feature map.

(1) $${F^{\prime }}_{n+1,n}={F^{\prime \prime }}_{n+1}⊕{F^{\prime }}_{n}$$

where ⊕ denotes the element-by-element summation operation.

Then, utilizing the features acquired in the previous step, the global and self-attentive relationship weights are calculated by the sigmoid function, respectively. As shown in Figure 3, the process shown in the upper part of the branch is the computation process of global attention features. We perform a two-dimensional global average adaptive pooling of the input features, and then use a convolutional kernel of size 1 × 1, and the channel dimension of output features is one-fourth of the channel dimension of input features to realize the dimensionality reduction of convolutional feature channels. In order to avoid the computed data being too large and the network over-fitting problem, we perform batch normalization and add modified linear units. Finally, the original count of channels for features is to be restored with a convolutional kernel of size 1 × 1, and batch normalization is performed to obtain global attention features.

The process shown in the lower branch is the computation process of local attention features. By adopting a 1 × 1 size convolution kernel, the channel dimension of the input features is minimized to one-fourth of the original size. Then, batch normalization is performed, and corrected linear units are added. Finally, the amount of original channels to which the channel dimension of the feature map is restored with a convolution kernel of size 1 × 1 is applied, and then all feature values are normalized to acquire self-attention features.

After summing the global attentional features and self-attentive features per element according to the corresponding positions, the sigmoid function is employed for computing the focused relationship parameters of the bottom layer in the adjacent feature layer, which is $S_{n+1,n}^{12}$. Similarly, the supplemental relationship parameter of the higher level is obtained, where $S_{n+1,n}^{11}=1-S_{n+1,n}^{12}$.

This leads to the focused relation feature map $S_{n+1,n}^{22}$ and the supplemental relation feature map $S_{n+1,n}^{21}$:

(2) $$S_{n+1,n}^{21}={F^{\prime \prime }}_{n+1}⊗S_{n+1,n}^{11}$$

(3) $$S_{n+1,n}^{22}={F^{\prime }}_{n}⊗S_{n+1,n}^{12}$$

where ⊗ indicates that the elements in the corresponding positions are calculated sequentially according to the multiplication rule. Finally, the complementary relationship feature map is obtained.

(4) $${F^{\prime \prime }}_{n}=S_{n+1,n}^{21}⊕S_{n+1,n}^{22}$$

By the same principle, we obtained the complementary relationship feature map for ${F^{\prime \prime }}_{1}$, ${F^{\prime \prime }}_{2}$, ${F^{\prime \prime }}_{3}$ and ${F^{\prime \prime }}_{4}$.

### 3.4. Enhanced Relationship Feature Map Calculation

Considering the main relationship feature maps of two neighboring layers, where one lower layer contains the contextual information of the upper layer and the main relationship feature map of the upper layer is a more abstract representation of the lower layer, there is a rich contextual dependency between these feature maps.

The purpose of this proposed section is to capture such contextual relationships for embedding into the higher-level feature maps of neighboring layers so as to enhance the representation of higher-level features.

The calculation process for the module is illustrated in Figure 4; let ${F^{\prime \prime }}_{n}\epsilon {\mathbb{R}}^{B\times C\times H_{n}\times W_{n}}$ denote the obtained primary relationship feature map, where B, C, $H_{n}$ and $W_{n}$ denote the number of learned features, the channel dimension of features, the horizontal dimension of features, and the vertical dimension of features in one training session, respectively.

To establish the high-level enhancement relationship between two adjacent layers of features ${F^{\prime \prime }}_{n}$ and ${F^{\prime \prime }}_{n+1}$, the GAP is calculated to acquire global feature map $Z_{GAP}\left({F^{\prime \prime }}_{n}\right)\epsilon {\mathbb{R}}^{C}$ and the GMP algorithm is utilized for local feature map $Z_{GMP}\left({F^{\prime \prime }}_{n}\right)\epsilon {\mathbb{R}}^{C}$.

(5) $$Z_{GAP}\left({F^{\prime \prime }}_{n}\right)=G_{pool}\left({F^{\prime \prime }}_{n}\right)=\frac{1}{H_{n}\times W_{n}}\sum_{i=1}^{H_{n}}\sum_{j=1}^{W_{n}}{F^{\prime \prime }}_{n}\left(i,j\right)$$

(6) $$Z_{GMP}\left({F^{\prime \prime }}_{n}\right)=G_{\max}\left({F^{\prime \prime }}_{n}\right)=Max\sum_{i=1}^{H_{n}}\sum_{j=1}^{W_{n}}{F^{\prime \prime }}_{n}\left(i,j\right)$$

where $G_{pool}$ indicates that after GAP calculation and $G_{\max}$ indicates that after GMP calculation.

Then, the two results are imported into the MLP separately.

(7) $$M_{GAP}\left({F^{\prime \prime }}_{n}\right)=W_{1}\left(W_{0}\left(Z_{GAP}\left({F^{\prime \prime }}_{n}\right)\right)\right)$$

(8) $$M_{GMP}\left({F^{\prime \prime }}_{n}\right)=W_{1}\left(W_{0}\left(Z_{GMP}\left({F^{\prime \prime }}_{n}\right)\right)\right)$$

where $W_{0}\epsilon {\mathbb{R}}^{C/r\times C}$ and $W_{1}\epsilon {\mathbb{R}}^{C\times C/r}$r represents the scaling ratio of the channel dimension. $W_{0}$ and $W_{1}$ are convolutional operations. In particular, the activation function ReLU comes right after $W_{0}$ to avoid over-fitting and speed up network convergence.

Then, the output from the multilayer perceptron $M_{GAP}$ $M_{GMP}$ is subjected to an element-wise summation operation, followed by a Sigmoid activation operation to generate the enhanced weights of the adjacent two layers of feature maps:

(9) $$M_{A}\left({F^{\prime \prime }}_{n}\right)=\sigma \left(M_{GAP}\left({F^{\prime \prime }}_{n}\right)⊕M_{GMP}\left({F^{\prime \prime }}_{n}\right)\right)$$

where σ denotes the Sigmoid function.

After calculating the augmented weights of the adjacent two layers of feature maps, we perform an elemental multiplication to calculate the mapping with feature augmentation:

(10) $$F_{n}^{L}=\{\begin{array}{c} {F^{\prime \prime }}_{n}\;\;\;\;\;\;\;\;\;\;\;\;\;\;\;\;\;\;\;\;\;\;\;n=1\\ M_{A}\left({F^{\prime \prime }}_{n}\right)⊗{F^{\prime \prime \prime }}_{n+1}\;\;\;\;\;n=2,3,4 \end{array}$$

where ⊗ denotes the element multiplication operation. The enhanced relationship feature maps $F_{1}^{L}$, $F_{2}^{L}$, $F_{3}^{L}$, and $F_{4}^{L}$ can be calculated from Equation (10).

### 3.5. Feature Fusion and Objective Recognition

The advanced enhancement features are fused using the concatenation function to generate the final multilevel enhanced relationship feature map.

(11) $$F=Concat\left[F_{1}^{L},F_{2}^{L},F_{3}^{L},F_{4}^{L}\right]$$

Then, after GAP calculation, the flattened feature is obtained by pulling the global average pooled features into a one-dimensional vector using the flatten function. Then, the flattened features are input to the fully connected layer. Finally, We use one-hot coding to represent N categories of remote sensing scene categories, where the true probability of a category is denoted as $y_{ij}$. The predicted probability $\hat{y_{ij}}$ of each of the N categories is obtained by inputting $Z_{1}$ into the Softmax Layer.

The loss distance between the true probability and the predicted probability is determined by using the loss function; the smaller the loss value, the more accurate the prediction:

(12) $$Loss=-\frac{1}{B}\sum_{i=1}^{B}\sum_{j=1}^{N}-y_{ij}\log\hat{y_{ij}}-\left(1-y_{ij}\right)\log\left(1-\hat{y_{ij}}\right)$$

where N represents the total number of scene objective categories to be recognized.

## 4. Experiments and Results

### 4.1. Experiment-Related Settings

#### 4.1.1. Datasets

To evaluate the recognition effect for CRABR-Net under different numbers of remote sensing scene categories and different amounts of remote sensing scene data, the proposed CRABR-Net is validated on the following three datasets.

**Figure 5:**
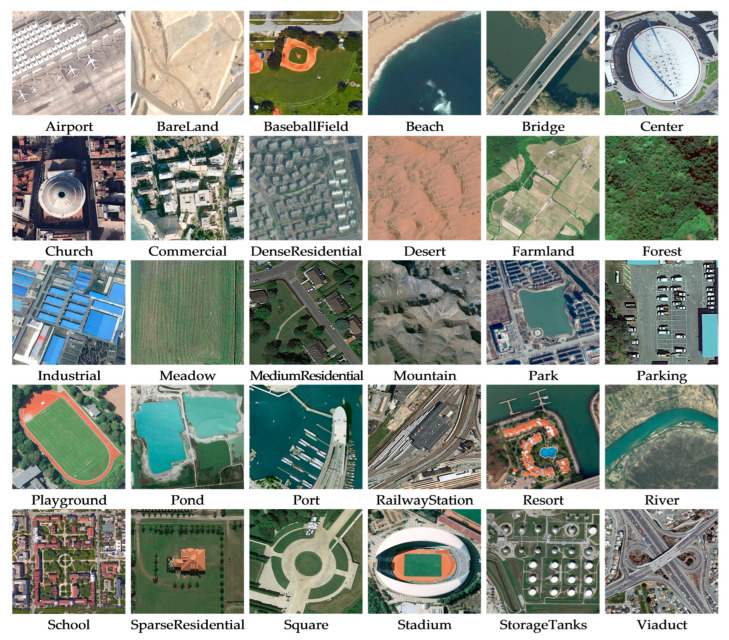
Instances of the scene objectives within AID Datasets.

**Figure 6:**
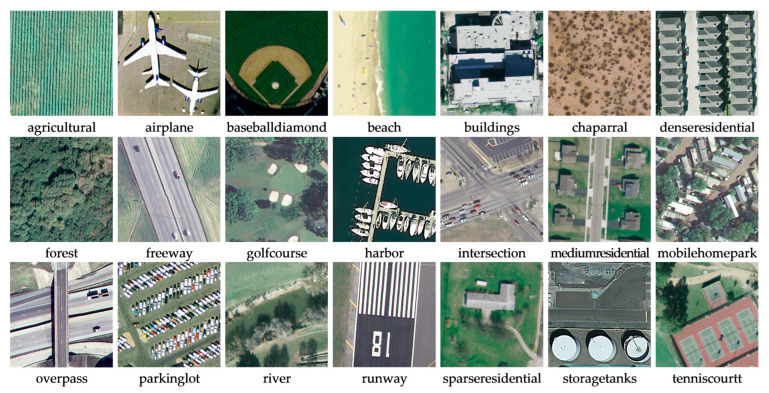
Instances of the scene objectives within UC-Merced Datasets.

**Figure 7:**
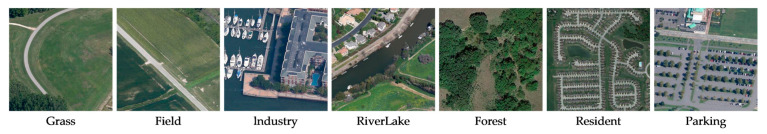
Instances of the scene objectives within RSSCN7 Datasets.

1. *AID Dataset*. It is a massive dataset of airborne scenes, acquired by collecting Google Earth images. It includes 30 categories of feature images of targets such as landforms, terrain, and buildings, and there are approximately 220 to 420 feature images collected for each category. The number of all images together is 10,000; in addition, the pixel size of each image is 600 × 600 [51]. Figure 5 shows instances of the scene objectives for every category within this dataset;
2. *UC-Merced Dataset*. It is an image data representing land use extracted manually by the researchers. These data reflect the land use within the city, and in terms of the main content reflected in the images, there are a total of 21 land use types, with 100 images of each type. The total number of images is 2100, and the size of each type of image is 256 × 256 [52]. Figure 6 shows instances of the scene objectives for every category within this dataset;
3. *RSSCN7 Dataset*. It is a typical scene target collected from Google Earth, acquired under the conditions of diverse seasonal changes and weather variations, and the data processing is challenging. It contains seven types of features, with a total of 400 images for each type of feature, where each image gets a size of about 400 × 400, for a total of 2800 images [53]. Figure 7 shows instances of the scene objectives for every category within this dataset.

#### 4.1.2. Experimental Environment Setup

Our work was performed on a Linux platform with four NVIDIA A100-type GPU processors installed. Considering the seamless use of NumPy and the ability to accelerate the training using GPUs, as well as the ability to use dynamic graph computation to make the network more flexible, we used PyTorch, a deep learning framework released by Facebook. In our proposed model, to fasten convergence and increase speed while reducing the over-fitting of the model, we used pretraining parameters, a distributed training approach, and take batch size to 64, using the Adam gradient function, set L2 regularization to 0.0001, set the learning rate to 0.0003, and trained the network with 200 Epochs.

#### 4.1.3. Data Preprocessing

To prove the advantages of the proposed method via comparative results, we borrowed ratios used by many previous most advanced algorithms in classifying the dataset during the experimental process. Specifically, for the UC-Merced dataset, we set the proportion of training data to verified data to 1:1 and 4:1, respectively, and for the AID dataset and RSSCN7 dataset, we set 1:4 and 1:1, respectively.

An insufficient amount of data can easily cause the model training results to be under-fitted. To minimize the possible adverse effects in this regard, we used a data enhancement technique from the image processing domain to generate new training samples for the data used in our experiments. Specifically, we further enhanced the data diversity using rotation, translation, and flip processing for all the data in the training set before feeding it into our proposed model, while the dimensions were all resized to 224 pixels × 224 pixels. In addition, we convert all data formats to tensor format and normalize them to facilitate data processing and ensure faster convergence when the program runs.

### 4.2. Performance Evaluation Metrics

To demonstrate the validity and sophistication of our proposed method CRABR-Net, we used several important evaluation metrics, namely Accuracy, Confusion Matrix (CM), Precision, Recall, and Specificity, to quantitatively evaluate.

#### 4.2.1. Accuracy

For the validation of the recognition performance with the model throughout the verified dataset, we calculated the recognition accuracy as follows:

(13) $$Accuracy=\frac{\sum_{i=1}^{N}\left(f\left(x_{i}\right)=y_{i}\right)}{N}$$

where the category of remote sensing scene sample $x_{i}$ is $y_{i}$, the overall amount of remote sensing scene objective is N, and the function of predicted category is f.

#### 4.2.2. Confusion Matrix

To determine which classes of samples the model misidentified and to obtain the probability of misidentifying samples in that class, we constructed CMs for the three datasets at different training ratios using PyTorch 3.7. The vertical coordinates represent the true category of the remote sensing scene objective, and the horizontal coordinates represent the categories identified by our method.

#### 4.2.3. Precision, Recall, Specificity

Precision, which indicates the accuracy rate, for the percentage of positive samples you predict that are identified correctly (i.e., identified the positive sample as a positive sample). The higher the precision, the more accurate the finding.

(14) $$Precision=\frac{T_{P}}{T_{P}+F_{P}}$$

where $T_{P}$ means identifying positive samples as positives, and $F_{P}$ means predicting negative samples as positives.

Recall is a metric of coverage, and the metric has multiple positive examples being divided into positives. The higher the recall, the more complete the search is.

(15) Recall=TPTP+FN

where $T_{P}$ means identifying positive samples as positive samples and $F_{N}$ means identifying positive samples as negative samples.

Specificity indicates the ability to predict negative cases (the higher, the better).

(16) $$Specificity=\frac{T_{N}}{T_{N}+F_{P}}$$

$T_{N}$ means identifying negative samples as negative samples.

### 4.3. Recognition Results

#### 4.3.1. Analysis of Accuracy

According to the characteristics of the proposed method, we chose three different methods of the same type to conduct a comparison experiment: single CNN, multiple CNNs, and CNN combined with AM, with the same proportion of training data, and analyzed the performance of CRABR-Net in three typical scenarios for accuracy. The specific comparison is given below:

Table 1 gives the results of scene objective recognition using CNNs for the AID dataset. Of the three datasets, the AID dataset is much more challenging because it has more sample classes and a larger number of samples. As shown in Table 1, among single CNNs, CaffeNet, GoogLeNet, and VGG-VD-16 all use the top-level features of CNNs for scene recognition, and VGG-16 combines pretraining parameters; among multi-CNNs, the literature [54] uses two deep networks to learn different features of the same data separately and uses the fused two depth features for scene recognition; the literature [55] fused local binary pattern features of remote sensing image data for classification; the literature [10] achieved scene recognition by tandem CNN network and CapsNet network; in CNNs combining AMs, Wang et al. [18] improved classification performance by using AM on top layer features to selectively focus on key regions; Sun et al. [56] used three layers of convolutional features to combine to form new features for scene recognition, and additionally added two auxiliary linear classifiers to promote network convergence; the literature [57] applied the self-attention mechanism and combined with SVM to achieve scene recognition. The CRABR-Net achieved an impressive performance in the scene recognition task; while utilizing 20% of the dataset for training, the accuracy obtained is about 94.02%, and utilizing 50% of the dataset for training, the accuracy obtained is about 96.46%.

The results of scene objectives recognition using CNNs for the UC-Merced dataset are given in Table 2. As shown in Table 2, two approaches are proposed in the literature [58]; one is to perform scene recognition using the fusion of feature maps from various convolutional layers, and the other is to continue collecting feature maps from various layers separately and then fuse them to perform scene recognition using the fused features. The CRABR-Net achieved impressive performance in the UC-Merced scene recognition task; while utilizing 50% of the dataset for training, the accuracy obtained is about 98.06%, and utilizing 80% of the dataset for training, the accuracy obtained is about 99.20%.

Table 3 gives the results of scene objective recognition using CNNs for the RSSCN7 dataset. In [59], scene recognition is achieved by fine-tuning the MobileNet V2 network and then using top-level features; Gao et al. [60] use channel attention and spatial attention to extract important information about features; in [61], a bilinear structure is built using deep separable convolution and regular convolution, to fuse feature of both branches for scene recognition; Liu et al. [62] proposes a weighted spatial pyramidal matching classification method based on collaborative representation. In [63], the features of each branch of the CaffeNet and the VGG-VD-16 network are fused separately, and then the features of both branches are fused to form new features for scene recognition; Xu et al. [64] use CNN and graph neural network in parallel to achieve scene recognition; As shown in Table 3, the CRABR-Net achieved impressive performance in the RSSCN7 scene recognition task, while utilizing 20% of the dataset for training, the accuracy obtained is about 93.21% and utilizing 50% of the dataset for training, the accuracy obtained is about 95.43%.

#### 4.3.2. Analysis of Confusion Matrix

To analyze the recognition accuracy of CRABR-Net for each sample category in the three datasets, we constructed prediction CM to demonstrate the performance, respectively.

**Figure 8:**
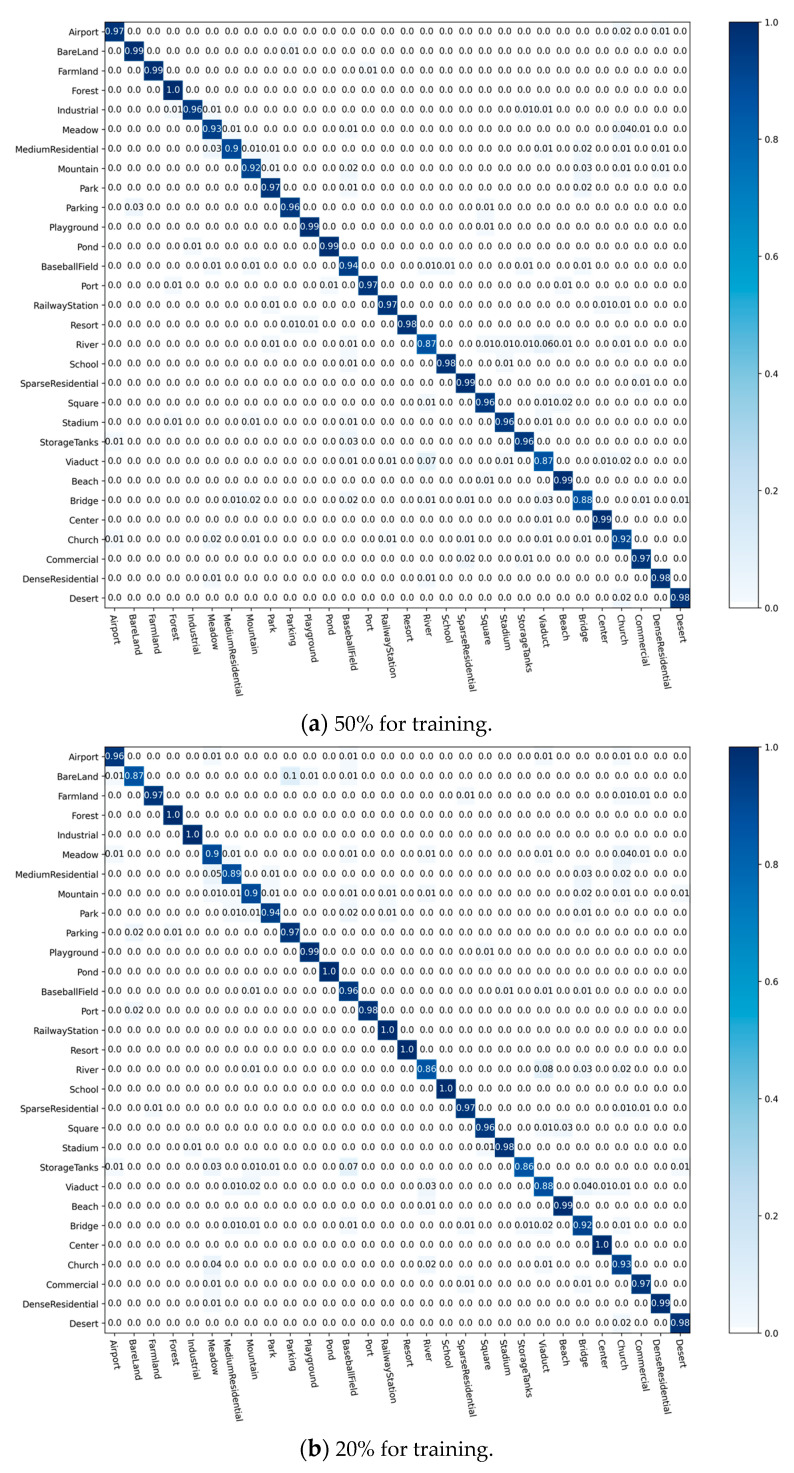
CMs on the AID dataset.

Figure 8 shows the CM generated under different proportions of AID training data. When the training data amount is 50% of all data, there are 27 remote sensing scene objective types recognized by our proposed method with an accuracy close to 100%; when the training data amount is 20% of all data, there are seven types recognized with 100% accuracy and eighteen types recognized with more than 90% accuracy; like “BareLand”, “MediumResidential”, “River”, “ StorageTanks”, “Viaduct”, and “Bridge” are difficult to recognize because of the large amount of overlap in the content of the image data, but despite this, our method achieves recognition accuracy of nearly 90%.

**Figure 9:**
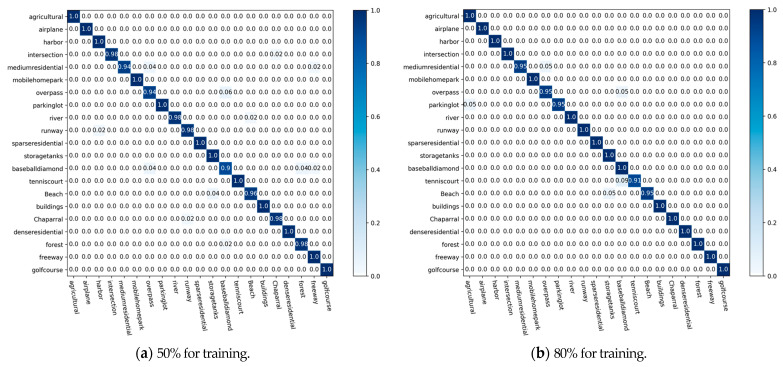
CMs on the UC-Merced dataset.

Figure 9 shows the CM generated under different proportions of UC-Merced training data. It is observed that all the types of remote sensing scene objectives are recognized by our proposed method with no less than 90% accuracy; 12 types are recognized with 100% accuracy when the training data amount is 50% of all data; 16 types are recognized with 100% accuracy when the training data amount is 80% of all data.

**Figure 10:**
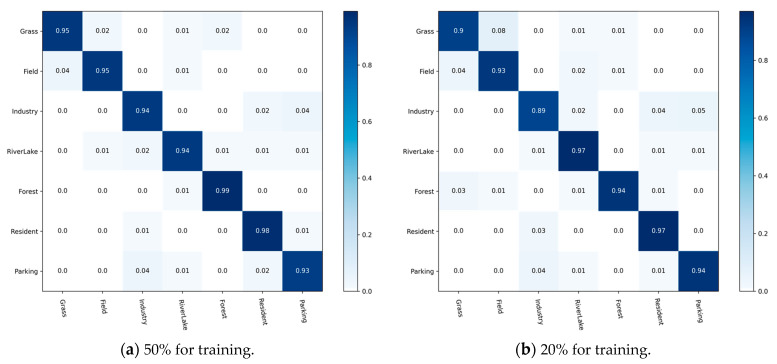
CMs on the RSSCN7 Dataset.

Figure 10 shows the CM generated with different proportions of RSSCN7 training data. It can be seen that because of the overlap between the contents of “Industry” and “Resident” and “Parking”, the accuracy of “Industry” is close to 90% when the training data accounts for 20% of the total data. When a percentage of up to 50% of the training data is increased, the accuracy of our proposed approach can be seen to be greater than 90% for all remote sensing scene objective types.

## 5. Discussion

To evaluate our proposed method scientifically, we have conducted sufficient ablation studies in three aspects: the typical model used in extracting features, the attention mechanism used in preprocessing, and the two modules used in the calculation of relational feature maps to verify the scientific validity of the present method.

### 5.1. Effects of Backbone Network

**Figure 11:**
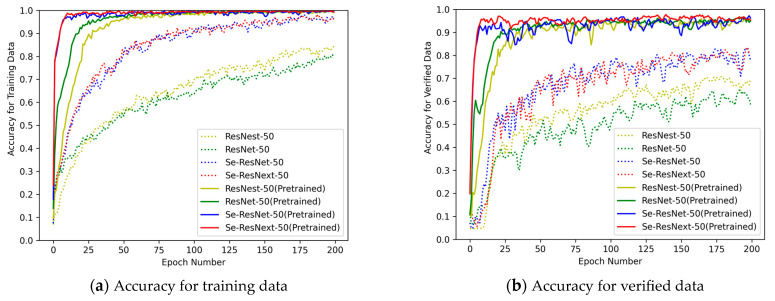
Accuracy on UC-Merced dataset.

For a better demonstration of how superior the Se-ResNext-50 model is in our proposed approach, we selected ResNet-50 and its improved model to compare the experimental effects. Specifically, the UC-Merced dataset is split into training data and validation data in a 1:1 ratio, at the same time keeping the feature preprocessing module and two relational feature calculation modules unchanged. In addition, the optimizer and learning rate, etc., were also kept unchanged, and only the backbone network for extracting features was replaced, and 200 epochs were trained to obtain the accuracy results of RSSOR, as shown in Figure 11.

The left panel in Figure 11 shows the recognition accuracy of different backbone networks in the training data, while the right panel shows the recognition accuracy of different backbone networks in the verified data. The solid line indicates that we used pretraining parameters in the training, and the dashed line indicates that we did not use pretraining parameters. Obviously, the Se-ResNext-50 model with pretraining parameters in the same case not only converges quickly and smoothly during the learning process in both datasets, but also has the highest target recognition accuracy. Therefore, it is clear that the convolutional network backbone model used has some superiority.

### 5.2. Effects of Attentional Mechanism

With the aim of analyzing the influence of various attention mechanisms in the feature preprocessing stage on the final recognition effect of our method, we selected three typical attention mechanisms containing Efficient Channel Attention (ECA) [65], Convolutional Block Attention Module (CBAM) [66], and SimAM, and conducted validation experiments with other conditions remaining the same and not being changed, respectively. The specific results are presented in Table 4.

Scientific and rational use of AM can improve the overall performance of the model to a certain stage on the basis of the original method performance level. To facilitate the comparison of experimental effects, we set up a new function, named maximumrate, to evaluate the enhancement of method performance by the attention mechanism by calculating the proportion of the amount of category scene features of the maximum evaluation metric. The bolded numbers in Table 4 indicate the maximum rate of the results in this category, and it is easy to discover from the Maximum rate that the best results were achieved when we used SimAM, both in precision, recall, and specificity of the samples. Conversely, when ECA and CBAM were used, the Maximum rate of the sample data was lower than when no attention mechanism was used. Therefore, we chose SimAM with facilitation in the preprocessing stage.

### 5.3. Effects of MLP, GAP, and GMP

In enhanced relationship feature map calculation, the number of feature channels input to the MLP is 256, so we set seven different scaling values, and using the UCM dataset trained under the same conditions, we obtained the accuracy of the model under different channel scaling ratios. As can be seen from Table 5, the model has the highest accuracy when the scaling ratio is equal to 16.

To verify the effect of GAP and GMP on the accuracy of the model, we designed three combinations and trained them under the same conditions, as shown in Table 6; when both GAP and GMP are involved in the training, the local enhancement coefficients and global enhancement coefficients of the input features are involved in the relationship enhancement computation, which leads to the highest accuracy of the model.

### 5.4. Effects of Feature Fusion Strategy

Towards analyzing the influence of multilevel enhancement relationship features on scene recognition effect under different fusion strategies, on the basis of fusing four-level features by using the concatenation function, we carried out comparison experiments on four high-level enhancement features according to the ways of fusing three-level features, fusing two level features and no fusing.

**Figure 12:**
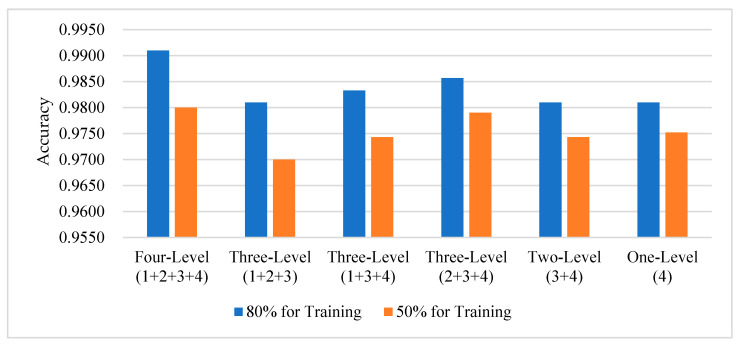
Accuracy for different feature fusion strategies.

We design the model architecture in each of the four different fusion methods according to the mathematical approach to combination. When no features are fused, the channel dimension is minimized, which is 256; when two features are fused, the channel dimension is 512; and when three features are fused, the channel dimension is 768. Using 80% and 50% of the UCM data, we train under the same conditions. Figure 12 lists some of the results of the experiments, from which it can be seen that the enhanced features are able to obtain high accuracy; in addition to the different strategies for combining the features, the recognition accuracy of the model under the same conditions is also different. When all four levels of features are concatenated by the concatenation function, the channel dimension reaches 1024, and the features at this time fully integrate the relationship information between the features at all levels, and after training, the model has the highest accuracy rate.

### 5.5. Effects of Calculation Module

For analyzing the effect of our proposed complementary relationship and enhanced relationship module on the recognition effect of the model, we set four different combinations of the relationship module under the same other conditions, so as to verify the recognition accuracy of the method in terms of different combinations of modules.

**Figure 13:**
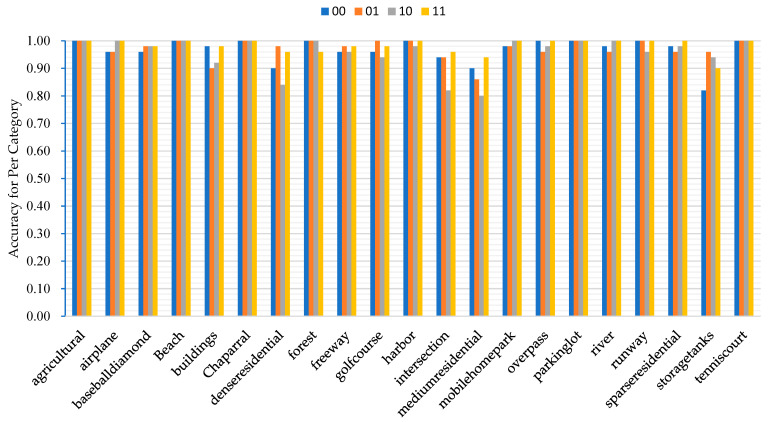
Accuracy for per category with different module combinations.

As shown in Figure 13, the “00” mode indicates that the complementary and augmented relationship modules are not used; the “01” mode indicates that the complementary relationship module is not utilized, but the augmented relationship module is utilized; the “10 “ mode indicates that the complementary relationship module is utilized and the augmented relationship module is not utilized; “11” mode indicates that the complementary relationship module and the augmented relationship module are utilized. We conducted comparison experiments on the UC-Merced dataset to obtain the recognition of each category of scene targets. From the figure, we can see that the “11” mode has relatively high accuracy and is more stable than the other modes.

## 6. Conclusions

Not only because of the complexity of remote sensing scene image data, but also because of the simple application of features to each layer of CNN, all of which affect the improvement of scene objective recognition accuracy to a certain extent. To solve the issue, we use the convolutional feature message of the upper layer to complement the lower layer, and complementary weights between adjacent layers are calculated using the self-attention relation and the global attention relation, and then the weights are assigned to the adjacent layers to complementary relationship feature maps, and the global and local features of the underlying layers are extracted to form the guide coefficients, and then fused with the features of the upper layers to obtain the enhanced relationship feature maps, and finally the features are fused to achieve scene objective recognition using softmax recognizer. The network is able to capture the key contents of scene objectives and enhance the representation of deep features by using the complementary relationships between contextual features and enhanced relational information, further improving the performance of scene recognition based on CNNs effectively. Experimental results on three common benchmark data collections (including AID, UC-Merced, and RSSCN7) indicate that CRABR-Net can fully utilize the powerful learning ability of CNN and realize higher recognition accuracy. In the next work, we will investigate various network architectures to enhance the efficiency of remote sensing scene objective recognition further by fusing and optimizing different networks.

## Figures and Tables

**Table 1:** Scene objective recognition accuracy on the AID dataset.

Modes	Solutions	Accuracy	
		20%	50%
○	CaffeNet [49]	86.86 ± 0.47	89.53 ± 0.31
	GoogLeNet [49]	83.44 ± 0.40	86.39 ± 0.55
	VGG-VD-16 [49]	86.59 ± 0.29	89.64 ± 0.36
	VGG-16(fine-tuning) [54]	89.49 ± 0.34	93.60 ± 0.64
◎	Two-Steam Fusion [55]	92.32 ± 0.41	94.58 ± 0.25
	TEX-Net-LF [56]	90.87 ± 0.11	92.96 ± 0.18
	VGG-16-CapsNet [10]	91.63 ± 0.19	94.74 ± 0.17
	Inception-v3-CapsNet [10]	93.79 ± 0.13	96.32 ± 0.12
●	GBNet [54]	90.16 ± 0.24	93.72 ± 0.34
	GBNet + global feature [54]	92.20 ± 0.23	95.48 ± 0.12
	AlexNet + SAFF [57]	87.51 ± 0.36	91.83 ± 0.27
	VGG_VD16 + SAFF [57]	90.28 ± 0.29	93.83 ± 0.28
	ARCNet-VGG16 [18]	88.75 ± 0.40	93.10 ± 0.55
Ours	CRABR-Net	94.02 ± 0.34	96.46 ± 0.23

○ for single CNN; ◎ for multiple CNNs; ● for CNN combined with AM.

**Table 2:** Scene Objective Recognition Accuracy on the UC-Merced Dataset.

Modes	Solutions	Accuracy	
		50%	80%
○	CaffeNet [49]	93.98 ± 0.67	95.02 ± 0.81
	GoogLeNet [49]	92.70 ± 0.60	94.31 ± 0.89
	VGG-VD-16 [49]	94.14 ± 0.69	95.21 ± 1.20
	VGG-16(fine-tuning) [54]	96.57 ± 0.38	97.14 ± 0.48
◎	Two-Steam Fusion [55]	96.97 ± 0.75	98.02 ± 1.03
	TEX-Net-LF [56]	95.89 ± 0.37	96.62 ± 0.49
	VGG-16-CapsNet [10]	95.33 ± 0.18	98.81 ± 0.22
	MSDS [58]	-	96.96 ± 0.84
	MLDS [58]	-	97.88 ± 0.71
●	ARCNet-VGG16 [18]	96.81 ± 0.14	99.12 ± 0.40
	HONGLIN WU [57]	95.81 ± 0.98	97.43 ± 0.94
	GBNet [54]	95.71 ± 0.19	96.90 ± 0.23
	GBNet+global feature [54]	97.05 ± 0.19	98.57 ± 0.48
	AlexNet + SAFF [57]	96.13 ± 0.97	-
	VGG_VD16 + SAFF [57]	97.02 ± 0.78	-
Ours	CRABR-Net	98.06 ± 0.24	99.20 ± 0.19

○ for single CNN; ◎ for multiple CNNs; ● for CNN combined with AM.

**Table 3:** Scene objective recognition accuracy on the RSSCN7 dataset.

Modes	Solutions	Accuracy	
		20%	50%
○	CaffeNet [49]	85.57 ± 0.95	88.25 ± 0.62
	GoogLeNet [49]	82.55 ± 1.11	85.84 ± 0.92
	VGG-VD-16 [49]	83.98 ± 0.87	87.18 ± 0.94
	Fine-turn MobileNet V2 [59]	89.04 ± 0.17	92.46 ± 0.66
◎	TEX-Net-LF [56]	92.45 ± 0.45	94.0 ± 0.55
	LCNN-BFF [61]	-	94.64 ± 0.21
	Yishu Liu [63]	-	92.37 ± 0.72
	DFAGCN [64]		94.14 ± 0.44
●	Yue Gao [60]	91.07 ± 0.65	93.25 ± 0.28
	Resnet+SPM-CRC [62]	-	93.86
	Resnet+WSPM-CRC [62]	-	93.90
	SE-MDPMNet [59]	92.65 ± 0.13	94.71 ± 0.15
Ours	CRABR-Net	93.21 ± 0.47	95.43 ± 0.79

○ for single CNN; ◎ for multiple CNNs; ● for CNN combined with AM.

**Table 4:** Model recognition effects under different attention mechanisms.

Class	Precision				Recall				Specificity			
	None	ECA	CBAM	SimAM	None	ECA	CBAM	SimAM	None	ECA	CBAM	SimAM
**Agricultural**	**1.0**	**1.0**	**1.0**	0.943	**1.0**	**1.0**	**1.0**	**1.0**	**1.0**	**1.0**	**1.0**	0.997
**Airplane**	**1.0**	**1.0**	**1.0**	**1.0**	**1.0**	0.96	**1.0**	**1.0**	**1.0**	**1.0**	**1.0**	**1.0**
**Baseball diamond**	**1.0**	0.925	0.98	**1.0**	0.98	0.98	**1.0**	0.98	**1.0**	0.996	0.999	**1.0**
**Beach**	**1.0**	**1.0**	**1.0**	**1.0**	0.98	**1.0**	**1.0**	**1.0**	**1.0**	**1.0**	**1.0**	**1.0**
**Buildings**	0.942	0.957	**0.98**	0.942	**0.98**	0.88	0.96	**0.98**	0.997	0.998	**0.999**	0.997
**Chaparral**	**1.0**	**1.0**	**1.0**	**1.0**	**1.0**	**1.0**	**1.0**	**1.0**	**1.0**	**1.0**	**1.0**	**1.0**
**Dense residential**	0.906	0.907	0.906	**0.923**	0.96	**0.98**	0.96	0.96	0.995	0.995	0.995	**0.996**
**Forest**	**1.0**	0.962	0.98	**1.0**	**1.0**	**1.0**	**1.0**	0.96	**1.0**	0.998	0.999	**1.0**
**Freeway**	0.962	0.942	0.98	**1.0**	**1.0**	0.98	0.98	0.98	0.998	0.997	0.999	**1.0**
**Golf course**	0.961	0.978	**1.0**	0.98	**0.98**	0.9	**0.98**	**0.98**	0.998	0.999	**1.0**	0.999
**Harbor**	**1.0**	**1.0**	**1.0**	**1.0**	**1.0**	**1.0**	**1.0**	**1.0**	**1.0**	**1.0**	**1.0**	**1.0**
**Intersection**	0.979	**1.0**	0.959	**1.0**	0.94	0.92	0.94	**0.96**	0.999	**1.0**	0.998	**1.0**
**Medium residential**	0.938	0.939	0.939	**0.959**	0.9	0.92	0.92	**0.94**	0.997	0.997	0.997	**0.998**
**Mobile home park**	0.98	0.98	0.98	**1.0**	**1.0**	**1.0**	**1.0**	**1.0**	0.999	0.999	0.999	**1.0**
**Overpass**	0.961	**1.0**	0.98	0.962	0.98	0.98	**1.0**	**1.0**	0.998	**1.0**	0.999	0.998
**Parking lot**	**1.0**	**1.0**	**1.0**	**1.0**	**1.0**	**1.0**	0.98	**1.0**	**1.0**	**1.0**	**1.0**	**1.0**
**River**	0.943	0.98	0.98	**1.0**	**1.0**	**1.0**	0.98	**1.0**	0.997	0.999	0.999	**1.0**
**Runway**	**1.0**	0.962	0.98	0.98	**1.0**	**1.0**	**1.0**	**1.0**	**1.0**	0.998	0.999	0.999
**Sparse residential**	**1.0**	0.98	**1.0**	0.962	0.98	0.96	0.98	**1.0**	**1.0**	0.999	**1.0**	0.998
**Storage tanks**	0.977	0.923	0.979	**1.0**	0.86	**0.96**	0.94	0.9	0.999	0.996	0.999	**1.0**
**Tennis court**	**1.0**	**1.0**	**1.0**	**1.0**	**1.0**	**1.0**	**1.0**	**1.0**	**1.0**	**1.0**	**1.0**	**1.0**
**Maximum rate**	52.38%	42.86%	47.62%	**71.43%**	61.90%	57.14%	57.14%	**76.19%**	52.38%	42.86%	47.62%	**71.43%**

**Table 5:** Accuracy at Different Ratio of MLP.

Scaling Ratio	r = 2	r = 4	r = 8	r = 16	r = 32	r = 64	r = 128
**Accuracy**	0.9771	0.9762	0.9781	0.98	0.9781	0.9752	0.9752

**Table 6:** Accuracy in Different Combinations of GAP and GMP.

GAP	GMP	MLP	Accuracy
×	√	√	0.9771
√	×	√	0.9781
√	√	√	0.98

× indicates no participation in the calculation.; √ indicates participation in the calculation.

## Data Availability

AID Dataset: https://pan.baidu.com/s/1mifOBv6#list/path=%2F (accessed on 20 June 2023); UC-Merced Dataset: http://weegee.vision.ucmerced.edu/datasets/landuse.html (accessed on 4 July 2023); RSSCN7 Dataset: https://pan.baidu.com/s/1slSn6Vz (accessed on 20 June 2023); The parameters of Se-ResNext-50: http://data.lip6.fr/cadene/pretrainedmodels (accessed on 21 June 2023).
